# Complement and T Cell Metabolism: Food for Thought

**DOI:** 10.20900/immunometab20190006

**Published:** 2019-06-20

**Authors:** Erin E. West, Claudia Kemper

**Affiliations:** 1 Laboratory of Molecular Immunology and Immunology Center, National Heart, Lung and Blood Institute, Bethesda, MD 20892, USA; 2 Faculty of Life Sciences and Medicine, King’s College London, London SE1 9RT, UK; 3 Institute for Systemic Inflammation Research, University of Lübeck, Lübeck 23562, Germany

**Keywords:** complosome, complement, T cells, CD46, intracellular complement, CD4 T cells, CD8 T cells, metabolism, CTL

## Abstract

The classical complement system is engrained in the mind of scientists and clinicians as a blood-operative key arm of innate immunity, critically required for the protection against invading pathogens. Recent work, however, has defined a novel and unexpected role for an intracellular complement system—the complosome—in the regulation of key metabolic events that underlie peripheral human T cell survival as well as the induction and cessation of their effector functions. This review summarizes the current knowledge about the emerging vital role of the complosome in T cell metabolism and discusses how viewing the evolution of the complement system from an “unconventional” vantage point could logically account for the development of its metabolic activities.

## Introduction

The complement system is generally considered among the evolutionary oldest parts of our immune system. It was discovered over a century ago by Jules Bordet as a liver-derived and serum-circulating system of proteins key to the detection and destruction of pathogens that have successfully breached the body’s protective epithelial borders [1]. Complement consists of over 50 proteins that either circulate in blood, the lymph and interstitial fluids, or are expressed on cells in mostly pro-enzyme and non-activated states. Sensing of pathogens or danger by one or more of the three activation pathways, the classical, the lectin, or the alternative complement pathway, triggers activation of the system in a cascade-like fashion. This culminates in the cleavage of the core complement effector molecules C3 and C5 into the bio-active anaphylatoxins C3a and C5a and the opsonins C3b and C5b. C5b-tagging of pathogens induces the formation of the lytic pore membrane attack complex (MAC) and direct killing of the microbe, whilst C3b mediates its opsonization and uptake by scavenger cells. The anaphylatoxins engage their respective G protein-coupled receptors (GPCR), the C3a receptor (C3aR) and the C5a receptor (C5aR1) on innate immune cells to induce their migration and activation to and at the site of pathogen breach [2–4]. Although complement was initially considered only a key constituent of innate immunity, due to its critical role in delivering co-stimulatory signals via engagement of complement receptors on antigen presenting cells (APCs) or direCTLy on B and T cells, it is now also widely recognized as a required functional bridge between innate and adaptive immunity [5–7].

Work on better defining the instructive role of complement on adaptive immune cells led to the somewhat surprising finding that these complement effects were mostly independent of liver-derived complement but rather mediated by locally produced and activated complement—for example, C3 and C5 secreted by APCs and then activated in the extracellular space [8–10]. The growing notion that compartmentalization of complement-mediated activity in immunity may exist was then supported by the discovery of an intracellularly generated and functioning complement system in human CD4^+^ and CD8^+^ T cells [11,12]. Likely the most exciting observation though about intracellular complement—coined the complosome to set it apart from the liver-derived and serum-circulating complement system [13]—is the finding that it unexpectedly serves key roles in single cell metabolism [12,14,15]. In this brief feature article, we give a succinct overview of our current understanding about the mechanistic roles of intracellular complement during the immunometabolic adaptions underlying the life cycle of human T cells. Further, and based on those new aspects of complement activity, we move into more uncharted areas and discuss a hypothetical alternative to the currently accepted model on how the complement system may have evolved and finally outline some of the key questions and challenges in this exciting new research area.

## The Complosome in Th1 Cell Biology

Metabolism plays an integral role in CD4^+^ T cell responses with naïve cells, the various effector cell subtypes (for example, T helper type (Th) 1, Th2, Th17 and regulatory T cells), and memory T cells each utilizing their own constellation of metabolic pathways, with differing dependences on influx/efflux of nutrients, their subsequent usage and the generation of downstream metabolic products. Excellent in-depth descriptions of the distinct metabolic programs used by T cell subpopulations can be found in many recent comprehensive reviews [16–18]. Here, we will focus more narrowly on an emerging area of immunometabolism; the role of complement and the complosome in human T cell metabolism.

The realization that an intracellular complement system exists and that it is an integral part of normal T cell biology is rooted largely in work surrounding the human-specific complement regulator and receptor CD46. CD46, also known as membrane cofactor protein (MCP) was initially identified as a transmembrane complement regulator that inhibits complement deposition on host cells by acting as a cofactor for the proteolytic cleavage and inactivation of C3b and C4b by the complement protease Factor I [19]. However, shortly after its discovery CD46 emerged as a multi-functional protein coordinating a broad range of activities on many cell types. Intriguingly, CD46 was identified to be a magnet for pathogens, acting as an entry receptor for a range of human disease-causing viruses and bacteria, including measles virus, herpesvirus, and Neisseria [20,21]. Additionally, CD46 also plays a role in the sperm-egg fusion, allowing for a normal acrosomal reaction [22–24]. Strong clinical interest in this molecule was triggered by the finding that mutations or single nucleotide polymorphisms (SNPs) in the *CD46* gene were either causative or contributing to diseases including hemolytic uremic syndrome and age-related macular degeneration [25–28]. Most recently— and the subject of this review—CD46 has been shown to play an integral and non-redundant role in human T cell responses, in large part by heavily influencing the metabolic state of these cells in the resting and/or activated phases [29,30].

While CD46 is expressed on all nucleated human cells, it is not expressed on rodent somatic cells, and thus represents a human specific pathway (discussed in more detail in a subsequent section)[23,31,32]. On human immune cells, CD46 is expressed in four distinct isoforms that differ in the level of *O*-glycosylation of the extracellular domains of the protein, and in their expression of two distinct intracellular tails, CYT-1 and CYT-2 (giving rise to CD46^CYT-1^ and CD46^CYT-2^ isoforms), that can signal and mediate discrete functions in a large spectrum of cell types [32,33]. However, although CD46 is ubiquitously expressed, CD46’s signaling pathways—as well as the complosome activities—have so far been best defined in CD4^+^ T cells.

**Figure 1:**
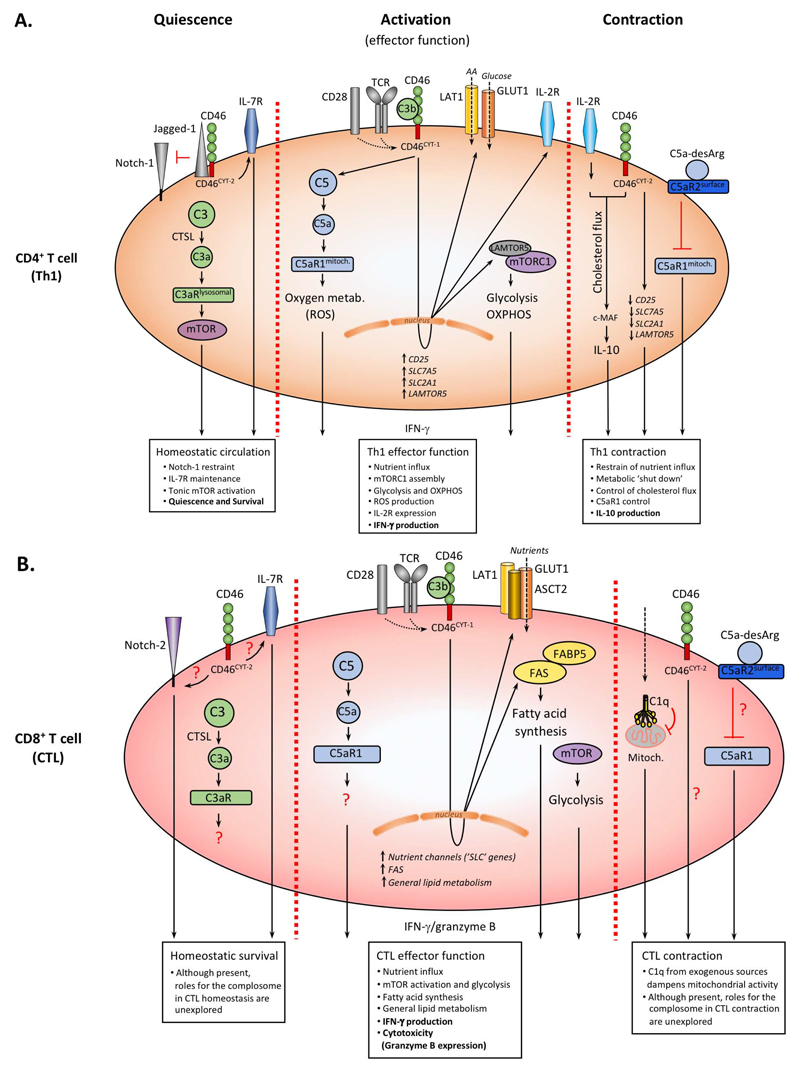
The Complosome in the “metabolic regulation” of Th1 and CTL responses. (**A**) Resting CD4^+^ T cells express predominantly the CD46^CYT-2^ form, which sustains IL-7R expression and restrains Notch signaling. CTSL-generated intracellular C3a engages lysosomally-expressed C3aR and drives tonic mTOR activation supporting homeostatic survival. TCR/CD28 activation induces autocrine CD46 engagement, a switch to CD46^CYT-1^ isoforms and nuclear translocation of γ-secretase-cleaved CYT-1 (not shown). This initiates increased GLUT1, LAT1, LAMTOR5, IL-2R expression, mTORC1 assembly, and high glycolysis and OXPHOS, whilst intracellular C5a drives ROS production further supporting Th1 activity. During Th1 contraction, CD46 and IL-2R initiate IL-10 co-induction which involves c-MAF expression and cholesterol flux, reversion to CD46^CYT-2^ isoforms, reduction in nutrient channel expression and autocrine surface C5aR2 engagement via C5a-desArg which suppresses intracellular C5aR1 signals. (**B**) Although present in circulating CTLs, a role for the complosome in CTL homeostasis and/or contraction remains unexplored (aside from the role of C1q in reduction of mitochondrial activity). During CTL activation, autocrine CD46 engagement induces nutrient transporter expression and nutrient influx with glycolysis induction and a particular increase in fatty acid synthesis. Impact on OXPHOS and oxygen metabolism by CD46 and/or intracellular C5a has not yet been formally assessed.

Serum-derived complement activity is usually connected with cell activating, inflammatory outcomes, in contrast, in quiescent T cells the complosome provides key metabolic signals that sustain cell survival while keeping the cell in the unactivated homeostatic state (Figure 1A). Intracellular low-level activation of C3 (either expressed by the T cell or taken up via the so-called C3 recycling pathway [34]) by cathepsin L provides key cell survival signals in “resting” T cells: intracellular C3a binds to C3aR1 on lysosomes resulting in tonic activation of the nutrient sensor mammalian target of rapamycin (mTOR) which allows for basic cell survival and glycolysis [13]. Additionally, C3b generated engages in an autocrine fashion CD46 isoforms predominantly expressing the “suppressive” CYT-2 tail. CD46^CYT-2^ drives interleukin (IL)-7 receptor (CD127) expression which sustains low level glucose transporter 1 (GLUT-1) surface levels. This in turn allows for glucose-mediated protein kinase B (AKT) activation and anti-apoptotic B cell lymphoma (BCL-2) expression, all events needed for cell survival [13,35–37]. Importantly, CD46 further maintains quiescent T cell homeostasis by preventing or restraining unwarranted Notch-1 activation, that would normally lead to T cell activation, by sequestering Jagged-1 away from Notch-1 [38](Figure 1A).

Whilst the complosome serves unexpected (metabolic) house-keeping functions in resting CD4^+^ T cells, it is also somewhat a “loaded gun” as activation of T cells via the T cell receptor (TCR) unleashes the “proinflammatory” potential of this system very rapidly: TCR stimulation induces increase of C3 activation and the shuttle of C3a and C3b to the cell surface. Autocrine CD46 stimulation by C3b then triggers the metalloproteinase-mediated cleavage of the surface portion of CD46 and subsequent release of the “brake” on Notch-1 [38–40]. TCR activation also induces upregulation of the “inflammatory” CD46^CYT-1^ isoform via a mechanism that is not understood yet. CD46^CYT-1^ mediated signals are absolutely required for IFN-γ production and Th1 induction, as patients deficient in CD46 or its ligand (C3/C3b) have severely reduced Th1 responses at least early in life and suffer from recurrent infections. Interestingly, both patient groups have normal Th2 and proliferative responses indicating that the complosome is particularly key to Th1 activity [38,41,42]. Further, autocrine CD46 and C3aR activation in CD4^+^ T cells is independent of serum-derived C3b, but can be fully driven by T cell-provided intra- and extracellular C3b and C3a generation [11,38,41]. Stimulation of CD46 not only induces surface processing of this molecule but simultaneously leads to γ-secretase-mediated cleavage of both intracellular tails. The tails translocate to the nucleus and this event allows for the CD46-mediated regulation of gene expression, of which many are metabolic sensors/regulators, enzymes and/or or nutrient transporters. For example, CD46^CYT-1^ is required for increased gene transcription of *SLC2A1*, coding for GLUT-1, of *SLC7A5*, coding for the large neutral amino acid transporter 1 (LAT-1) and of *LAMTOR5* (late endosomal/lysosomal adaptor, MAPK and MTOR activator 5 (LAMTOR5) is a scaffolding protein that supports mammalian target of rapamycin complex 1 (mTORC1) assembly at the lysosomes)). These events culminate in the high levels of glycolysis, amino acid influx and mTORC1 assembly and activation that are particularly needed for metabolically highly demanding IFN-γ and Th1 responses [14,43]. In parallel to the direct impact on the cell metabolic machinery, autocrine CD46^CYT-1^ signaling also results in increased expression of IL-2Ra (CD25) and assembly of the high affinity IL-2 receptor, necessary for optimal Th1 responses [6,44,45].

We recently also observed that human CD4^+^ T cells contain storages of intracellular C5 and generate low level C5a in the resting state. The enzyme that cleaves C5 into C5a remains to be defined though [15]. TCR triggering in conjunction with CD46 coactivation amplifies intracellular C5a generation which results in increased intracellular C5aR1 signaling from the mitochondria and the augmented production of reactive oxygen species (ROS). This intracellular ROS production initiates the assembly of a canonical NLR family pyrin domain containing 3 protein (NLRP3) inflammasome and secretion of mature IL-1β, which further maintains IFN-γ secretion and hence regulates the duration of Th1 responses in tissues [15](Figure 1A).

As important as a rapid induction of Th1 responses is to prevent pathogen invasion, the timely shut-down and resolution of such T cell effector activity is equally critical to the host’s health because it limits the pathological consequences of an over-exuberant or prolonged response [46–48]. This is elegantly demonstrated by the observation that mice deficient in the *Il10* gene can clear some infections more rapidly through strong Th1 immunity compared to wild type animals, but then succumb to uncontrolled tissue pathology as the anti-inflammatory cytokine IL-10 is key in limiting inflammatory pathology [47]. CD46, together with signals from the IL-2 receptor orchestrates Th1 contraction via the co-induction of IL-10 in Th1 cells once sufficient IFN-γ production and Th1-derived IL-2 levels are established (Figure 1A). The exact signals downstream of the IL-2R or CD46 that drive IL-10 production are not well-defined but it is understood that a reversion of the CD46 isoforms back to a predominant CD46^CYT-2^ form is required [11,14,49]. These signals lead to a general shut-down of the effector Th1 cell metabolic signature, by decreasing IL-2 signals, through the reduction of CD25 expression, and limiting nutrient influx, by downregulation of GLUT1, LAT1 and LAMTOR5 which cumulates in reduced mTORC1 activity and the general return of the cell to a metabolically resting state [14,30]. A recent paper shed some light on how CD46 induces IL-10 production via connecting CD46 activity with the regulation sterol metabolism: This study by Perucha and colleagues demonstrated a role for CD46 in the induction of the cholesterol biosynthesis pathway and normal cholesterol flux that is required for c-MAF-driven IL-10 expression in contracting Th1 cells [50](Figure 1A). Importantly, the intracellular C5 system also plays a part in this general complosome-controlled “shut-down” process as increased C5a-desArg production observed during Th1 expansion engages the inhibitory C5aR2 in an autocrine fashion and leads to a reduction in ROS generation and NLRP3 inflammasome activation [15](Figure 1A). The exact mechanism, as to how C5aR2 controls this process is currently not defined.

In sum, the complosome is an integral component of the metabolic signatures that denote Th1 homeostasis, effector function induction and contraction and with this partakes in all phases of the complete “Th1 life cycle”.

## The Complosome and CTL Activity

The integral role of metabolism in CD8^+^ T cell function has been appreciated for some time and continues to be a feverous area of research [16–18,51]—much driven by the exciting advances in and growing success of tumor-specific CD8^+^ T cell adoptive transfers for cancer treatment [52]. The functional intersection of complement and CD8^+^ T cell biology/metabolism, however, is just beginning to be appreciated and represents currently a largely unexplored research area. We do, however, expect this to change rapidly and the limited work that is published allow a glimpse into likely important roles for complement and the complosome in the positive and negative control of human CD8^+^ cytotoxic T cells (CTLs).

Human CD8^+^ T cells, similar to CD4^+^ T cells, also express all of the complosome components, including stores of C3 and C5 and their cognate complement receptors C3aR1, C5aR1 and C5aR2, and CD46, thus setting the stage for a role of the complosome in CD8^+^ T cell responses. TCR and CD28 stimulation increases complosome activation and leads to autocrine engagement of CD46 also in these T cells (Figure 1B). However, and in contrast to CD4^+^ T cells, where CD46 signals are absolutely required for IFN-γ production and Th1 induction, in CD8^+^ T cells CD46 signals are not obligatory but rather support optimal CTL effector activity induced by TCR and CD28 engagement [12,53]. More specifically, in human CD8^+^ T cells, CD46 costimulation augments both CTL effector cytokine secretion (IFN-γ and TNF-α) and their ability to kill target cells, with concomitant increases in degranulation and granzyme B secretion. The CD46^CYT-1^ and CD46^CYT-2^ isoform expression pattern in resting and activated CD8^+^ T cells parallels that of CD4^+^ T cells, however, their distinct contributions to CTL induction and possibly contraction have not been defined. The supportive role of CD46 during CTL effector induction is mediated through enhancement of amino acid influx (e.g., increased expression of the heterodimeric amino acid transporter LAT-1) which supports increased mTOR activation and glycolysis. Importantly, defining the role of CD46 during CTL stimulation allowed us to identify a novel role for the complosome in fatty acid metabolism, as CD46 drives the high levels of fatty acid synthase (FASN) and fatty acid binding protein 5 (FABP5) expression needed for sustained CTL activity (Figure 1B). A somewhat puzzling finding is the observation that CD46 triggers the activation of the intracellular C5 system as well as NLRP3 expression also in human CD8^+^ T cells, but that canonical NLPR3 inflammasome activity and intrinsic IL-1β production seem to not be required for normal IFN-γ secretion and CTL activity—this is in stark contrast to CD4^+^ T cells where both systems are key to functional Th1 activity [12]. Thus, while the normal production of their signature cytokine IFN-γ in both Th1 and CTL cells requires the involvement of the complosome, there are clearly specific differences in the (metabolic) pathways triggered by the complosome between these T cell subpopulations. This is in essence not surprising as it is broadly acknowledged in the field that even within the CD4^+^ T cell compartment, different subsets (for example, Th1, Th2, Treg, or Th17 cells) display distinct metabolic requirements, amino acid usages, and dependence on glycolysis and OXPHOS for their appropriate functional repertoires [16–18,54].

Although homeostatic survival of naïve human CD8^+^ T cells also requires IL-7 receptor mediated signals [55] and the expression of Notch-2 is known to control the generation, survival and function of CD8^+^ T cells [56], the potential contributions of CD46 to IL-7R expression or its engagement with the Notch system to support CD8^+^ T cell homeostasis have not been assessed (Figure 1B). Similarly, a role for CD46 or other complosome components during CD8^+^ T cell contraction remains to be established. Of note, one study suggests that CD46 fails to drive IL-10 in CTLs, further underpinning differences in complosome activities between CD4^+^ and CD8^+^ T cells [53]. Nonetheless, there is indication that intracellular complement can, analogously to what is observed in CD4^+^ T cells, also exert negative control over CD8^+^ T cells to dampen CTL responses and prevent immunopathologies. Ling *et al*. demonstrated that C1q, taken up from an exogenous source by human CD8^+^ T cells, engages the intracellular C1q receptor p32/gC1qR expressed on mitochondria and induces a reduction in mitochondrial activity. This translates into a general negative control of CTL activity with decreased proliferation, survival and cytotoxic activity. In consequence, C1q deficiency results in exacerbated CTL responses in mouse models of autoimmunity graft versus host disease (GVHD) and viral infection [57]. The authors further show that the lack of C1q, as present in C1q-deficient patients that all develop systemic lupus erythematosus (SLE), contributes to uncontrolled CTL activity, which then propagates the autoimmune disease state in these patients.

In summary, the complosome emerges as a “master regulator” of the transporter systems that control nutrient (in)flux and of the enzymes that shunt (incoming) nutrients into the correct metabolic adaption pathways required for appropriately catered T cell activities towards sensed antigen and/or incoming environmental cues.

## The Complosome in Mice and Men

Most of the studies on the complosome so far have utilized purified CD4^+^ T cells derived from healthy donors and pertinent patient groups and then analyzed *ex vivo* or *in vitro*. Although this has been an informative approach on several levels, it does not allow to define the role of the complosome in the context of the whole organism. Studies aiming to better understand the *in vivo* roles of the complosome in T cell metabolism in health and disease using small animal models face the sizeable hurdle that, although complement activity is needed for normal T cell immunity in both mice and men, there are substantial differences in complosome composition and function between these species. For example, whilst expression of C3aR and C5aR1 and 2 by human CD4^+^ T is considered well established [12,15], their presence in mouse T cells is hotly debated. Three independent studies using reporter animals have failed observe C3ar, or C5ar1 or 2 expression on resting or activated T cells [58–60] while some other groups detected expression of these receptors on murine T cells [9,61]. Of note, when detected, C5ar2 seems to only appear on the mouse T cell surface after activation [62], in contrast to human T cells where C5aR2 is constitutively found on the cell surface in both resting and activated cells.

More importantly, the critical driver of metabolic reprogramming in human Th1 cells, CD46, is not found on somatic tissue in rodents as these species only express a “membrane cofactor protein” (gene: *Cd46*) in immune-privileged tissues such as the eye and the inner acrosomal membrane of spermatozoa [31,63,64]. Also, the composition of the mouse *Cd46* gene and the resulting protein does not support the expression of different isoforms nor the processing of its single intracellular domain—which bears no homology to either CYT-1 or CYT-2 of human CD46—by the γ-secretase complex, further supporting that there is little relation with human CD46 [32]. A functional homologue within the complement family that mimics the activity of human CD46 and would integrate an autocrine role for C3b during T cell stimulation in mice is currently not defined, and the resulting lack of a suitable small animal model hampers our efforts to conclusively probe the *in vivo* roles of CD46. It is not clear as to why the mouse genome did not sustain CD46 expression during evolution. One hypothetical—although intuitive—possibility is, that mice aimed to protect themselves against infections with CD46-binding pathogens, a strategy successfully adopted by New World monkeys who modified their CD46 structure in such way that it retained complement regulative activity but lost the measles virus binding site [23]. Humans, however, may have retained CD46 expression because its central role in host cell metabolism and homeostasis may still outweigh the “cost” of an increased risk of infections. Similarly, we can currently only speculate about what exact path rodent cells took to regulate the metabolic pathways controlled by CD46 in humans. Although not formally proven, we champion the notion that Notch may have taken on CD46’s role in mice. This view is based on the early evolutionary co-appearance of the Notch and complement systems [30], the close functional relationship between Jagged/Notch and CD46 in the regulation of human CD4^+^ T cell activity (Figure 1A; [38]), the intriguing structural similarities between Notch and CD46 (activation-induced sequential processing by ADAMs and γ-secretase and nuclear translocation of cytoplasmic signaling domains) and the fact that Notch regulates the same metabolic pathways, including glycolysis, OXPHOS and fatty acid metabolism [65,66]. The outcome of such Notch-regulated metabolic events also clearly align with the role of CD46 in CTL biology and Th1 induction and contraction: Notch is needed to induce IFN-γ secretion in human CD4^+^ and CD8^+^ T cells [56,67] but also drives IL-10 co-expression in Th1 cells [68]. It should be noted that a large proportion of experiments surrounding *in vitro* studies about Notch’s role in human T cell biology include the usage of γ-secretase inhibitors. This treatment not only inhibits Notch but also inadvertently inhibits CD46 processing and function and hence precludes an unequivocal assignment of observed metabolic effects solely to Notch inhibition.

Studying human-specific complement and metabolic pathways remains challenging. However, technological advances in system approaches with regards to systematically screening large human-based data sets for disease-gene discovery [69,70] and applying increasingly sensitive whole-metabolome analyses [71] in combination with a focused effort to better define and understand the breadth of pathologies in patients with inborn errors of metabolism [72] hold the promise for substantial future progress in this exciting research area.

## Complement Evolution—A Revised View

**Figure 2:**
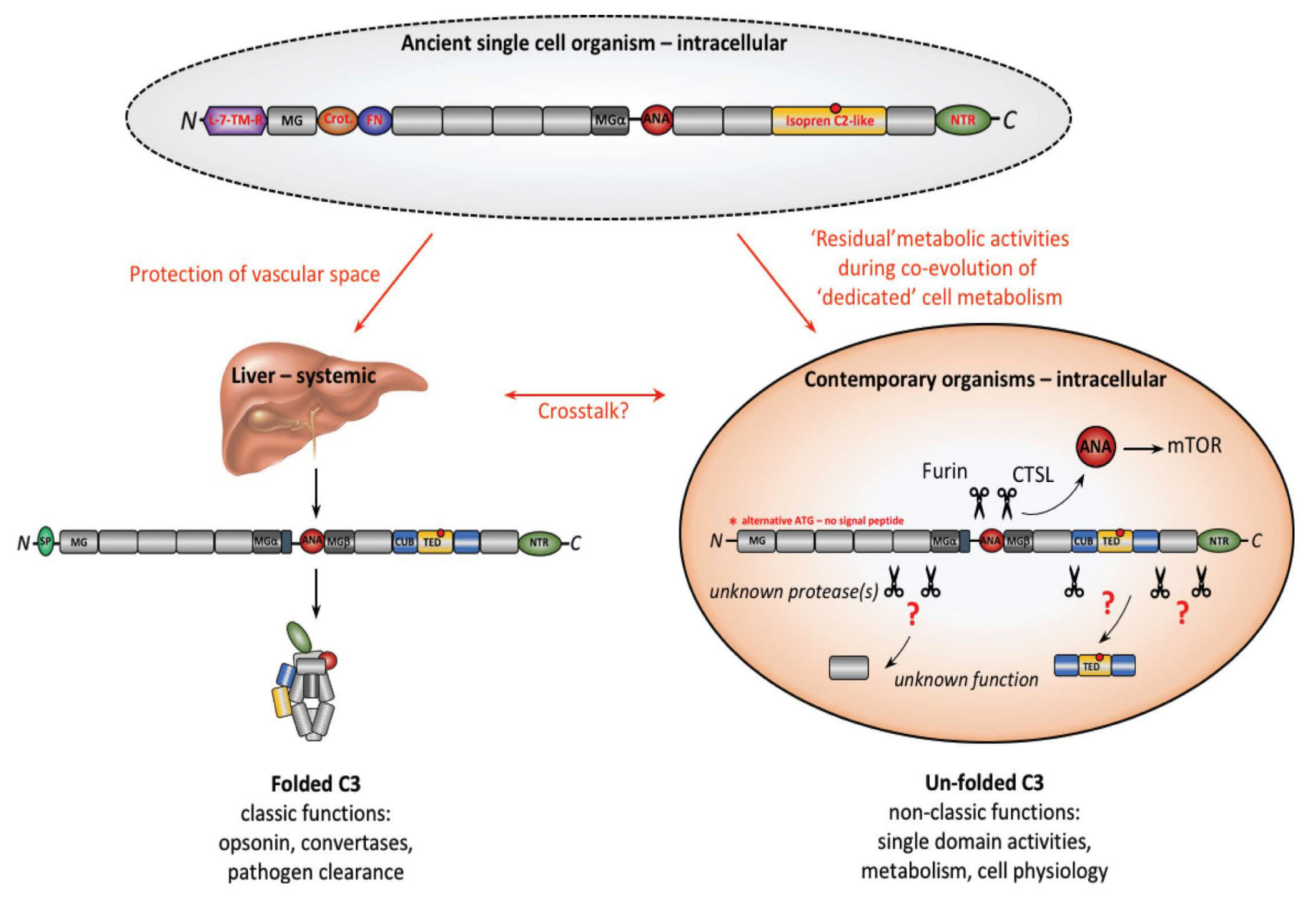
Proposed evolution of extra- and intracellular C3. Evolutionary oldest C3 (shown is C3 combining domains found in tunicates, fish, birds, and early mammals) contained additional domains with metabolic activities [29] and may appeared first in ancient single cell organisms where it regulated cell physiology. During evolution of life into multi-organ organisms, contemporary complement diverged into two principle “arms”: the liver-derived, systemic system that today protects the host’s vascular space against pathogens through “classically” folded and activated C3 (opsonin and C3/C5 convertase activity and MAC induction) and intracellular C3 still serving key functions in metabolism. “Modern” intracellular C3 likely lost metabolic domains due to concurrent co-evolution of a “dedicated” cell metabolism machinery. The discovery of intracellular C3 transcribed from an alternative ATG and lacking a signal peptide supports this idea [87]. Further, intracellular C3 may not acquire the classic C3 folded structure but is rather processed by cell-specific proteases to activate domains needed for metabolism—as previously shown for C3a (ANA). Thus, other C3 domains (freed by yet unknown proteases) could also serve new functions in basic cell physiology. ANA, anaphylatoxin dom.; crot., crotonase dom.; CUB, complement C1r/C1s, Uegf, Bmp1 dom.; FN, ferredoxin reductase dom.; L-7-TM-R, 7-lung-transmembrane dom.; MG, macroglobulin-like dom.; NTR, *N*-terminal region-like dom. TED, thioester containing dom. (red circle).

Metabolic activity is the defining characteristic of life and with the complement system being among the evolutionary oldest parts of our immune system [29,73], a close functional complement-metabolism relationship could somewhat be anticipated. C3 is considered not only the core effector molecule but also the origin of the complement system [74]. And as a matter of fact, very early and now also recent work showed that C3aR-mediated signals on adipocytes or pancreatic β-cells may impact on their energy usage, hinting that C3 has an indeed tight connection with metabolism [30,75–77]. However, most functional considerations of C3 have so far been based on the contemporary C3 form that is secreted by the liver as a complex folded structure and that, when activated, functions as source for C3a, as the opsonin C3b, and component of C3 and C5 convertases (Figure 2)—and a direct connection with single-cell metabolism is hence not obvious. However, a closer look into the C3 forms of the evolutionary oldest organisms revealed that they often contained distinct additional protein domains that displayed a high homology with metabolically-active molecules [29] and that have been lost in higher vertebrate C3 (Figure 2). For example, tunicate C3 harbored a crotonase (Crot) domain which is a defining feature of members of the crotonase/Enoyl-Coenzyme A (CoA) hydratase superfamily, a critical driver of the β-oxidation of fatty acids [78]. The FN domain retained within C3 of birds can be traced back to the ferredoxin NADP(H) reductase (FNR) superfamily members found already in ancient anaerobic bacteria and cyanobacteria [79]. Ferrodoxin NADP(H) reductase was originally identified as driver of electron transfer from in the electron transport chain (ET) mechanism of the Photosystem I during photosynthesis in plants [80], whilst FNR functions in as electron transporter in mitochondria and regulator of sterol, cholesterol and steroid metabolism in humans [81]. The Boreoeutheria, considered early predecessors of primates, contained a 7-lung-transmembrane domain in their C3 form. This domain is similar to early GPCR-proteins now only retained in some amoebozoa, desmosponges, and invertebrates [82] and was functionally essential for the coordination of intracellular vesicular transport and proglucagon mRNA synthesis. Of note, “metabolic” domains can actually still be observed in modern C3 of humans: an isoprene C2-like domain which is contained in proteins regulating cholesterol metabolism [83] is “buried” in the CUB (complement C1r/C1s, Uegf, Bmp1) and TED (thioester containing) domains. Similarly, the C3 *N*-terminal region (NTR)-like domain is found in molecules that orchestrate cell migration during neural development [84] but also in tissue inhibitors of metalloproteinases (TIMPs), key regulators of extracellular matrix turnover [85].

Based on the conceptually fitting structural evolution of C3 and the key role of the complosome in single cell metabolism, we had previously suggested that complement may have appeared originally as intracellular system, sensing and controlling the metabolic states of single-cell organisms and this idea is gaining traction in the field [14,29,30,86]. With the evolution of life into multi-organ organisms, there may have occurred *compartmentalization of complement function* over time: part of modern C3 remained active mostly intracellularly with a “functional eye” largely on single cell physiology—but may have lost some metabolic domains due to parallel development of an increasingly complex and dedicated metabolic machinery. A recent observation by King *et al*., who found that C3 in pancreatic β-cells is transcribed from an alternative ATG start site leading to a purely intracellularly retained protein due to lack of a “secretory” signal peptide [87] is in support of this notion. In line with the idea that cell-derived C3 has distinctive features from liver-derived C3, we have found that post-translational modifications of C3 differ between intracellular C3 and liver-derived C3 (unpublished data). With the emergence of tissue formation and lymphatic/interstitial/vascular systems, C3, in parallel, also specialized into a second direction as a secreted and systemic danger sensing system with opsonic and killing activity and into the guardian of our extracellular space as it is recognized for over a century now. Such bi-furcated model of C3 development opens the door to another intriguing updated concept that we are currently embracing in the laboratory: while circulating C3 needs to assume the “classically” folded protein form to function, upon activation, as opsonin, as the building block of the C3/C5 convertases, and to seed the formation of the lytic membrane attack complex, we suggest that the *intracellular C3 form does not need to acquire the classic folded C3 structure* for its activities (Figure 2). Intracellular C3 may rather be processed by cell-specific proteases to release and/or activate the domains needed for its cell metabolic activities from an unfolded mature protein or even pre-cursor C3 form—as it is the case for the anaphylatoxin C3a that can be generated by cathepsin L-mediated cleavage from a furin-processed C3 molecule within cells [11]. Under such concept, other C3 domains (for example, the CUB/TED domains that are at this moment considered structural support elements only) could be processed by a currently unknown protease and may serve an additional metabolic function (likely in cholesterol metabolism, see above) during T cell activation. This model suggestion, although exciting, is speculative at this point and needs to be probed for validity in the future. Tracing back the evolution of C3 and also other complement proteins in not yet precisely DNA and RNA-sequenced organisms on a broad level could indeed deliver new insights into the functional connection between the complosome and cell physiology.

## Summary/Outlook

Recent published work strongly advocates that complement activity within T cells is among the key regulators of normal cell metabolism and induction of effector function. Given our current understanding of the complosome, it is likely that intracellular complement also partakes in the generation of T cell memory and/or tissue residency, subjects that we are currently exploring. Furthermore, although the complosome has so far been mostly studied in T cells, it is present in a broad range of cells [11], and we therefore expect that future research will discover and define additional new and critical roles for this system in cell physiology. In line with his notion is the recent finding that intracellular C3 regulates autophagy and survival of stressed pancreatic β-cells [87].

The modulation of metabolic pathways in immune cells for therapeutic application, specifically in cancer, is currently a hot topic. The complosome may provide a valuable new pharmacological target but in order to pursue this with a prospect of success, we first need to acquire a better understanding of its activity. For example, so far, we know very little about the subcellular localization of the distinct complosome components, how their activation is induced and controlled by cells and/or the environment, and how the complosome composition looks like and functions in other cells where it is clearly observed. Also, the cross-talk of the complosome with liver-derived complement, which surely exists, is an entirely unexplored area and needs to be understood. Addressing these important questions, though, is not straight forward. Aside from the differences in complosome functions between mice and man as discussed above, it is now broadly acknowledged that almost all complement receptors function in a cell-specific fashion—hence requiring the generation of cell-specific KO animals for the assessment of each cell type of interest. Further, complement components often operate in a bi-phasic mode, contributing during both the cell activation (inflammatory) and “cessation” phases (anti-inflammatory)[15,88,89], thus, one ideally wants to employ inducible and cell-specific *in vitro* and *in vivo* systems. And, of course, detailed functional and mechanistic dissections of pathways controlling (T) cell metabolism have their own difficulties: for example, the study of T cell populations *ex vivo* under controlled nutrient and/or metabolic conditions is an important initial approach but often does not reflect the “metabolic behavior” of T cells during effector responses *in vivo*—which occur in tissues and embedded into a network of surrounding cells that impact heavily on T cell activity [90]. *In vivo* models, however, make it almost impossible to control for compensatory metabolic pathways that cells tend to engage in when faced with the engineered hypo- or hyper-activity of key metabolic nodes, which is most well characterized in cancer cells [54,91].

However, there is rapid progress in key technological advances and the field is currently generating many of the reagents required to better understand the new and exciting role of complement in “single cell” physiology. We hope that this review transports our continuous enthusiasm for this ancient immune sensor system and gives the reader some “food for thought” as to how it may integrate into their own (metabolic) research focus.
